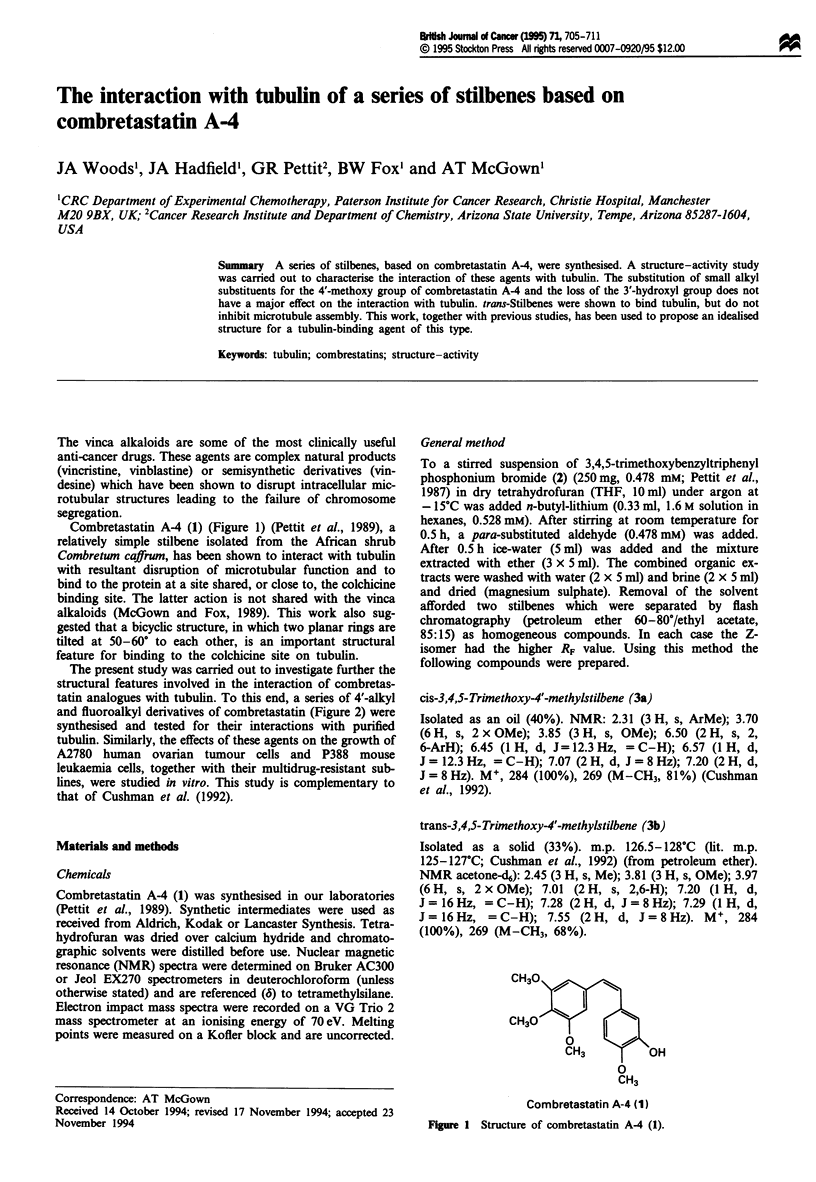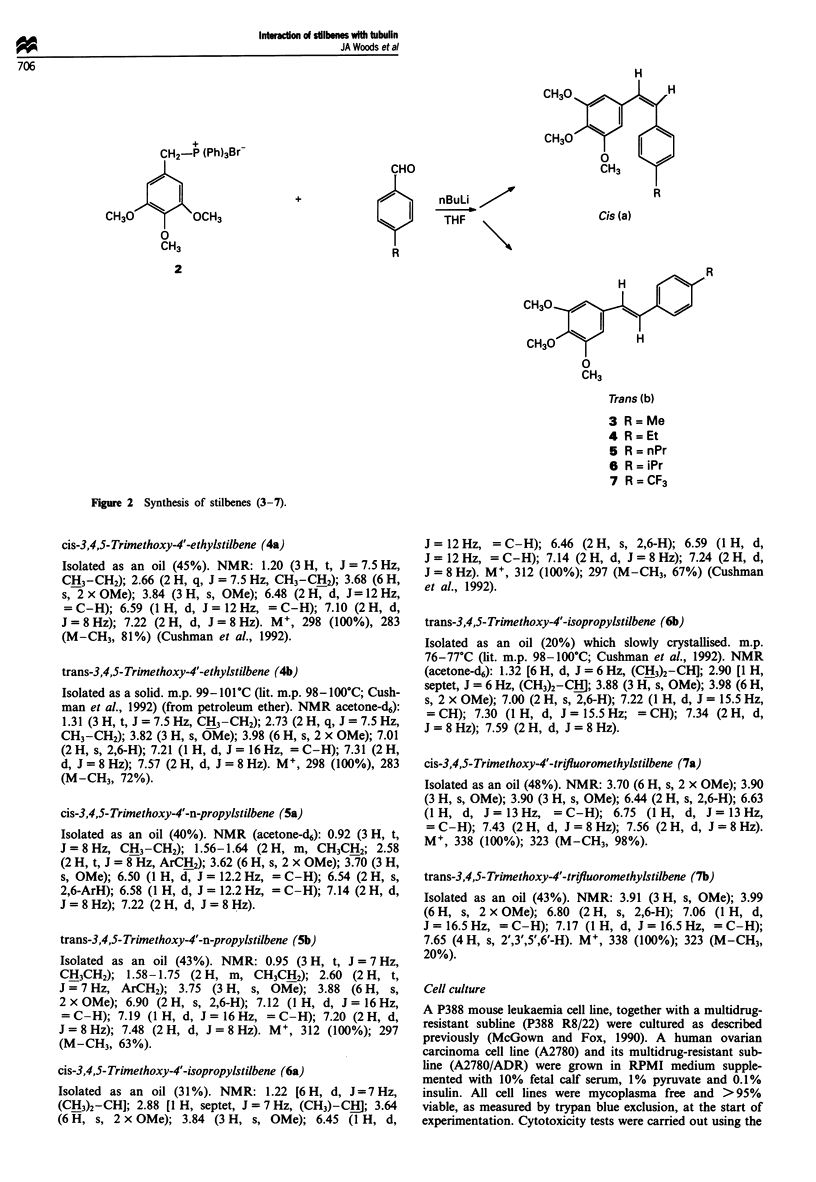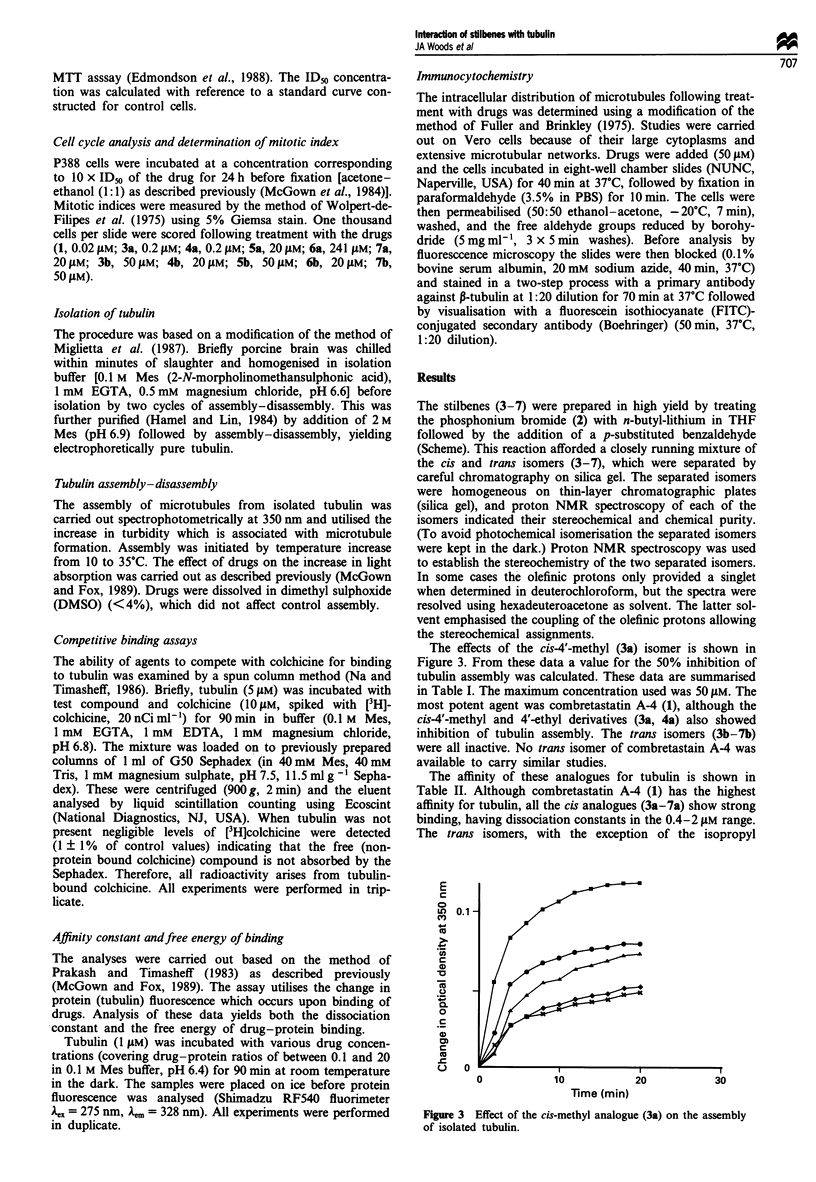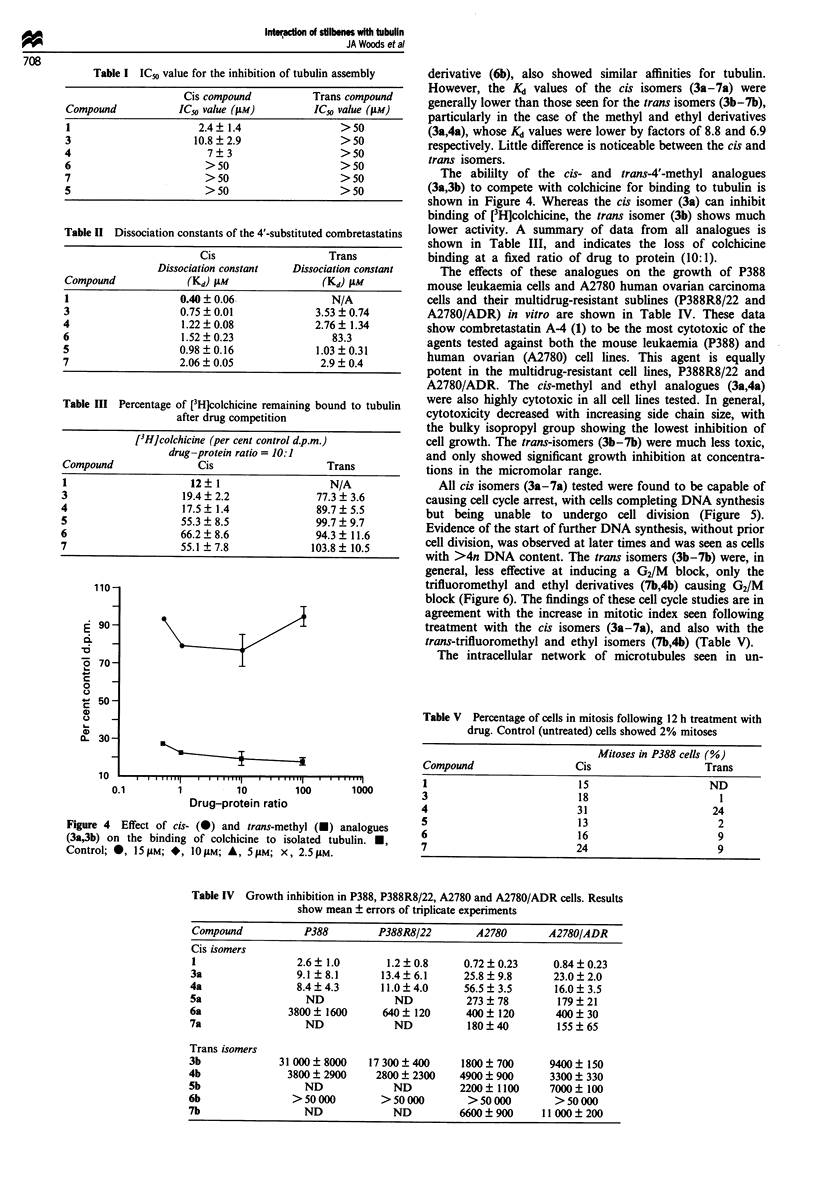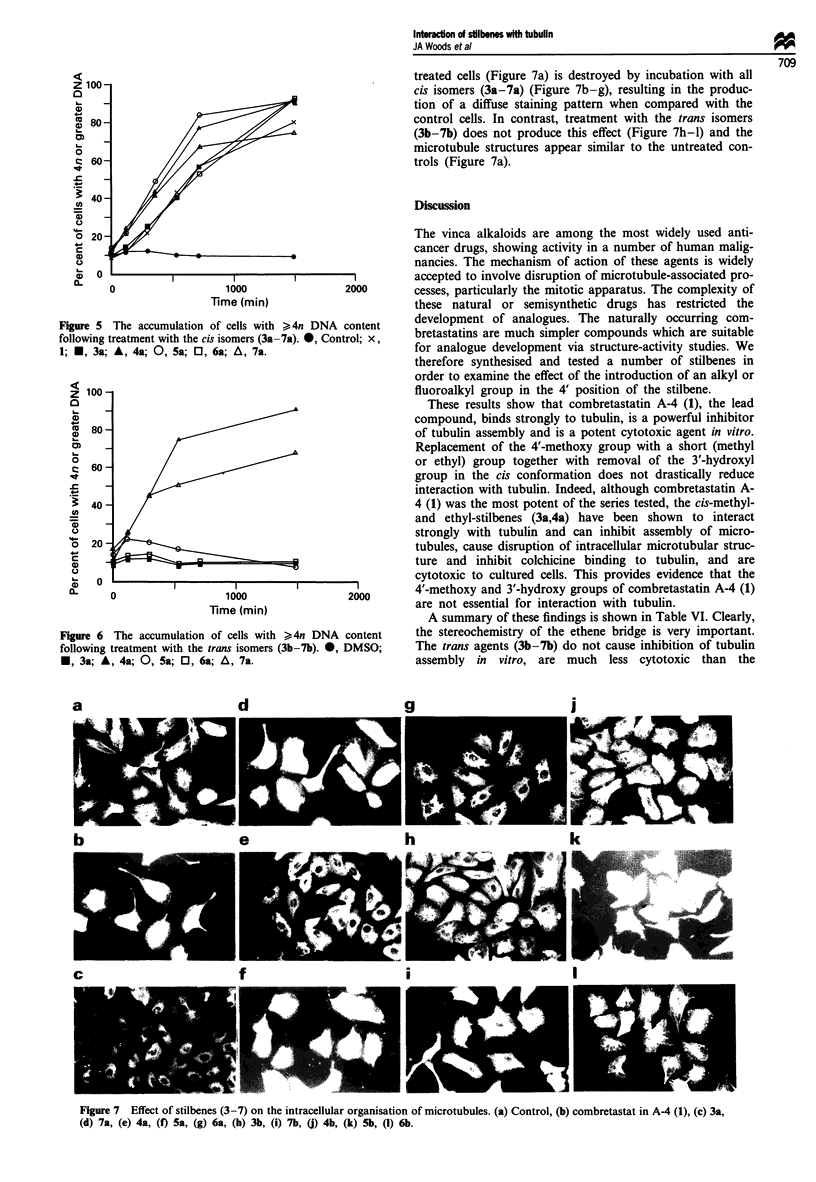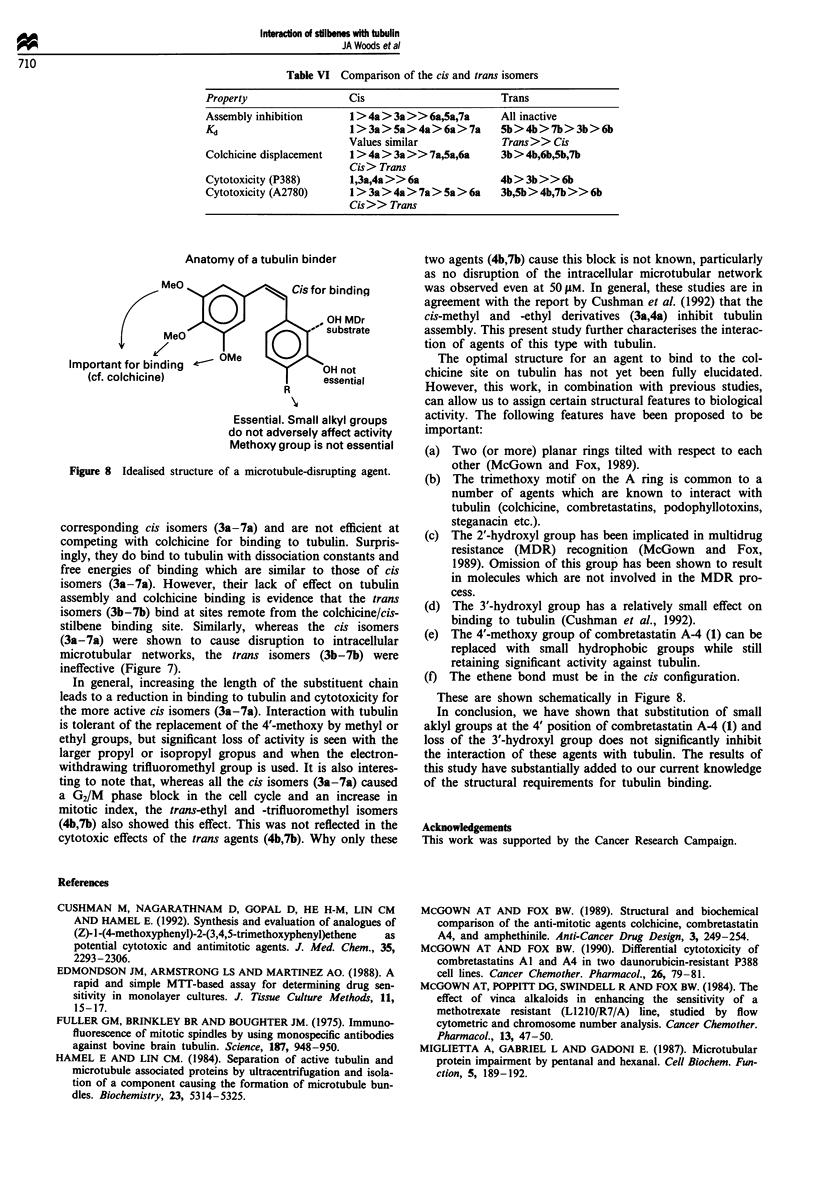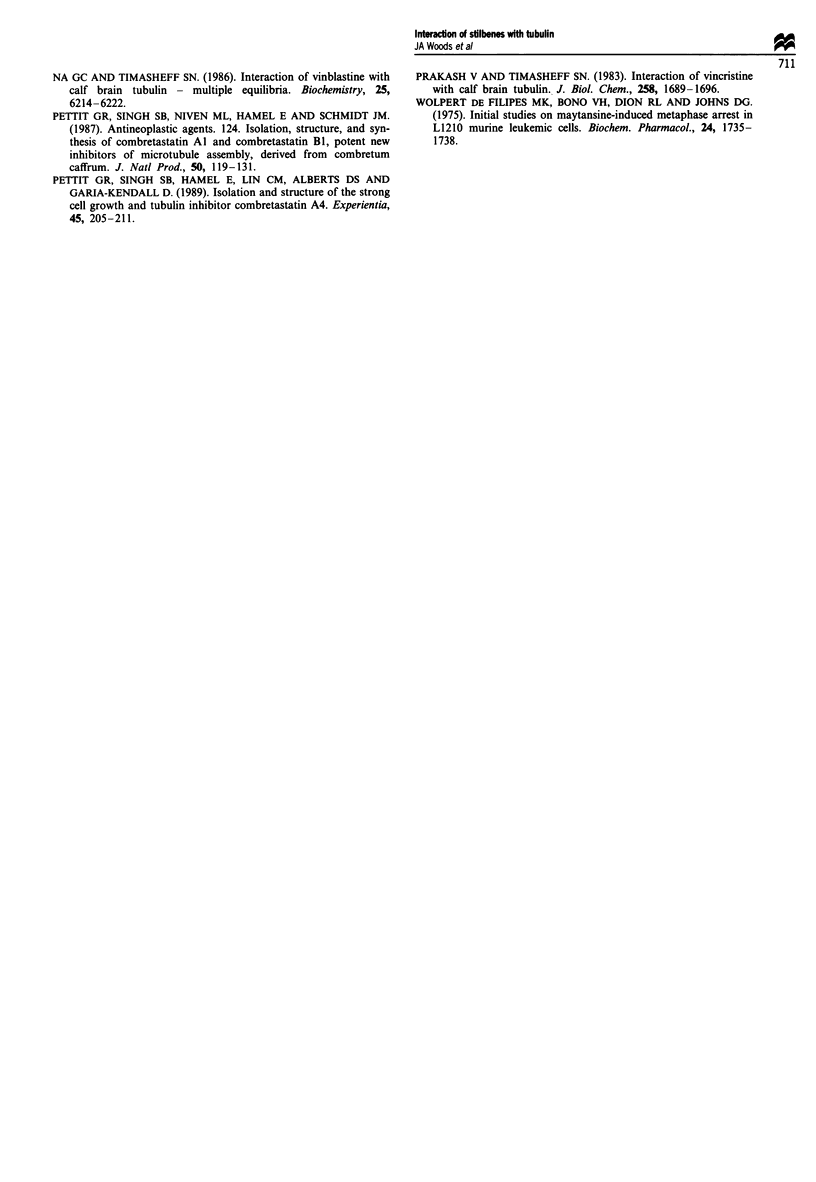# The interaction with tubulin of a series of stilbenes based on combretastatin A-4.

**DOI:** 10.1038/bjc.1995.138

**Published:** 1995-04

**Authors:** J. A. Woods, J. A. Hadfield, G. R. Pettit, B. W. Fox, A. T. McGown

**Affiliations:** CRC Department of Experimental Chemotherapy, Paterson Institute for Cancer Research, Christie Hospital, Manchester, UK.

## Abstract

**Images:**


British Journal of Cancer (1995) 71, 705-711

© 1995 Stockton Press All rights reserved 0007-0920/95 $12.00

### The interaction with tubulin of a series of stilbenes based on combretastatin A-4

JA Woods[1], JA Hadfield[1], GR Pettit[2], BW Fox[1] and AT McGown[1]

[1]*CRC Department of Experimental Chemotherapy, Paterson Institute for Cancer Research, Christie Hospital, Manchester M20 9BX, UK;* [2]*Cancer Research Institute and Department of Chemistry, Arizona State University, Tempe, Arizona 85287-1604, USA*

**Summary** A series of stilbenes, based on combretastatin A-4, were synthesised. A structure–activity study was carried out to characterise the interaction of these agents with tubulin. The substitution of small alkyl substituents for the 4'-methoxy group of combretastatin A-4 and the loss of the 3'-hydroxyl group does not have a major effect on the interaction with tubulin. *trans*-Stilbenes were shown to bind tubulin, but do not inhibit microtubule assembly. This work, together with previous studies, has been used to propose an idealised structure for a tubulin-binding agent of this type.

**Keywords**: tubulin; combrestatins; structure–activity

The vinca alkaloids are some of the most clinically useful anti-cancer drugs. These agents are complex natural products (vincristine, vinblastine) or semisynthetic derivatives (vindesine) which have been shown to disrupt intracellular microtubular structures leading to the failure of chromosome segregation.

Combretastatin A-4 (**1**) (Figure 1) (Pettit *et al.*, 1989), a relatively simple stilbene isolated from the African shrub *Combretum caffrum*, has been shown to interact with tubulin with resultant disruption of microtubular function and to bind to the protein at a site shared, or close to, the colchicine binding site. The latter action is not shared with the vinca alkaloids (McGown and Fox, 1989). This work also suggested that a bicyclic structure, in which two planar rings are tilted at 50–60° to each other, is an important structural feature for binding to the colchicine site on tubulin.

The present study was carried out to investigate further the structural features involved in the interaction of combretastatin analogues with tubulin. To this end, a series of 4'-alkyl and fluoroalkyl derivatives of combretastatin (Figure 2) were synthesised and tested for their interactions with purified tubulin. Similarly, the effects of these agents on the growth of A2780 human ovarian tumour cells and P388 mouse leukaemia cells, together with their multidrug-resistant sublines, were studied *in vitro*. This study is complementary to that of Cushman *et al.* (1992).

### Materials and methods

### Chemicals

Combretastatin A-4 (**1**) was synthesised in our laboratories (Pettit *et al.*, 1989). Synthetic intermediates were used as received from Aldrich, Kodak or Lancaster Synthesis. Tetrahydrofuran was dried over calcium hydride and chromatographic solvents were distilled before use. Nuclear magnetic resonance (NMR) spectra were determined on Bruker AC300 or Jeol EX270 spectrometers in deuterochloroform (unless otherwise stated) and are referenced (δ) to tetramethylsilane. Electron impact mass spectra were recorded on a VG Trio 2 mass spectrometer at an ionising energy of 70 eV. Melting points were measured on a Kofler block and are uncorrected.

Correspondence: AT McGown
Received 14 October 1994; revised 17 November 1994; accepted 23 November 1994

### General method

To a stirred suspension of 3,4,5-trimethoxybenzyltriphenyl phosphonium bromide (**2**) (250 mg, 0.478 mM; Pettit *et al.*, 1987) in dry tetrahydrofuran (THF, 10 ml) under argon at − 15°C was added *n*-butyl-lithium (0.33 ml, 1.6 M solution in hexanes, 0.528 mM). After stirring at room temperature for 0.5 h, a *para*-substituted aldehyde (0.478 mM) was added. After 0.5 h ice-water (5 ml) was added and the mixture extracted with ether (3 × 5 ml). The combined organic extracts were washed with water (2 × 5 ml) and brine (2 × 5 ml) and dried (magnesium sulphate). Removal of the solvent afforded two stilbenes which were separated by flash chromatography (petroleum ether 60–80°/ethyl acetate, 85:15) as homogeneous compounds. In each case the Z-isomer had the higher $R_F$ value. Using this method the following compounds were prepared.

### cis-*3,4,5-Trimethoxy-4'-methylstilbene (**3a**)*

Isolated as an oil (40%). NMR: 2.31 (3 H, s, ArMe); 3.70 (6 H, s, 2 × OMe); 3.85 (3 H, s, OMe); 6.50 (2 H, s, 2, 6-ArH); 6.45 (1 H, d, J = 12.3 Hz, = C–H); 6.57 (1 H, d, J = 12.3 Hz, = C–H); 7.07 (2 H, d, J = 8 Hz); 7.20 (2 H, d, J = 8 Hz). $M^+$, 284 (100%), 269 (M–$CH_3$, 81%) (Cushman *et al.*, 1992).

### trans-*3,4,5-Trimethoxy-4'-methylstilbene (**3b**)*

Isolated as a solid (33%). m.p. 126.5–128°C (lit. m.p. 125–127°C; Cushman *et al.*, 1992) (from petroleum ether). NMR acetone-$d_6$): 2.45 (3 H, s, Me); 3.81 (3 H, s, OMe); 3.97 (6 H, s, 2 × OMe); 7.01 (2 H, s, 2,6-H); 7.20 (1 H, d, J = 16 Hz, = C–H); 7.28 (2 H, d, J = 8 Hz); 7.29 (1 H, d, J = 16 Hz, = C–H); 7.55 (2 H, d, J = 8 Hz). $M^+$, 284 (100%), 269 (M–$CH_3$, 68%).

Combretastatin A-4 (**1**)

**Figure 1** Structure of combretastatin A-4 (**1**).

*Trans* (b)

**3** R = Me
**4** R = Et
**5** R = nPr
**6** R = iPr
**7** R = $CF_3$

**Figure 2** Synthesis of stilbenes (3–7).

cis-*3,4,5-Trimethoxy-4′-ethylstilbene (***4a***)*

Isolated as an oil (45%). NMR: 1.20 (3 H, t, J = 7.5 Hz, $CH_3$–$CH_2$); 2.66 (2 H, q, J = 7.5 Hz, $CH_3$–$CH_2$); 3.68 (6 H, s, 2 × OMe); 3.84 (3 H, s, OMe); 6.48 (2 H, d, J = 12 Hz, = C–H); 6.59 (1 H, d, J = 12 Hz, = C–H); 7.10 (2 H, d, J = 8 Hz); 7.22 (2 H, d, J = 8 Hz). $M^+$, 298 (100%), 283 (M–$CH_3$, 81%) (Cushman *et al.*, 1992).

trans-*3,4,5-Trimethoxy-4′-ethylstilbene (***4b***)*

Isolated as a solid. m.p. 99–101°C (lit. m.p. 98–100°C; Cushman *et al.*, 1992) (from petroleum ether). NMR acetone-$d_6$): 1.31 (3 H, t, J = 7.5 Hz, $CH_3$–$CH_2$); 2.73 (2 H, q, J = 7.5 Hz, $CH_3$–$CH_2$); 3.82 (3 H, s, OMe); 3.98 (6 H, s, 2 × OMe); 7.01 (2 H, s, 2,6-H); 7.21 (1 H, d, J = 16 Hz, = C–H); 7.31 (2 H, d, J = 8 Hz); 7.57 (2 H, d, J = 8 Hz). $M^+$, 298 (100%), 283 (M–$CH_3$, 72%).

cis-*3,4,5-Trimethoxy-4′-*n*-propylstilbene (***5a***)*

Isolated as an oil (40%). NMR (acetone-$d_6$): 0.92 (3 H, t, J = 8 Hz, $CH_3$–$CH_2$); 1.56–1.64 (2 H, m, $CH_3CH_2$; 2.58 (2 H, t, J = 8 Hz, Ar$CH_2$); 3.62 (6 H, s, 2 × OMe); 3.70 (3 H, s, OMe); 6.50 (1 H, d, J = 12.2 Hz, = C–H); 6.54 (2 H, s, 2,6-ArH); 6.58 (1 H, d, J = 12.2 Hz, = C–H); 7.14 (2 H, d, J = 8 Hz); 7.22 (2 H, d, J = 8 Hz).

trans-*3,4,5-Trimethoxy-4′-*n*-propylstilbene (***5b***)*

Isolated as an oil (43%). NMR: 0.95 (3 H, t, J = 7 Hz, $CH_3CH_2$); 1.58–1.75 (2 H, m, $CH_3CH_2$); 2.60 (2 H, t, J = 7 Hz, Ar$CH_2$); 3.75 (3 H, s, OMe); 3.88 (6 H, s, 2 × OMe); 6.90 (2 H, s, 2,6-H); 7.12 (1 H, d, J = 16 Hz, = C–H); 7.19 (1 H, d, J = 16 Hz, = C–H); 7.20 (2 H, d, J = 8 Hz); 7.48 (2 H, d, J = 8 Hz). $M^+$, 312 (100%); 297 (M–$CH_3$, 63%).

cis-*3,4,5-Trimethoxy-4′-isopropylstilbene (***6a***)*

Isolated as an oil (31%). NMR: 1.22 [6 H, d, J = 7 Hz, $(CH_3)_2$–CH]; 2.88 [1 H, septet, J = 7 Hz, $(CH_3)$–CH]; 3.64 (6 H, s, 2 × OMe); 3.84 (3 H, s, OMe); 6.45 (1 H, d, J = 12 Hz, = C–H); 6.46 (2 H, s, 2,6-H); 6.59 (1 H, d, J = 12 Hz, = C–H); 7.14 (2 H, d, J = 8 Hz); 7.24 (2 H, d, J = 8 Hz). $M^+$, 312 (100%); 297 (M–$CH_3$, 67%) (Cushman *et al.*, 1992).

trans-*3,4,5-Trimethoxy-4′-isopropylstilbene (***6b***)*

Isolated as an oil (20%) which slowly crystallised. m.p. 76–77°C (lit. m.p. 98–100°C; Cushman *et al.*, 1992). NMR (acetone-$d_6$): 1.32 [6 H, d, J = 6 Hz, $(CH_3)_2$–CH]; 2.90 [1 H, septet, J = 6 Hz, $(CH_3)_2$–CH]; 3.88 (3 H, s, OMe); 3.98 (6 H, s, 2 × OMe); 7.00 (2 H, s, 2,6-H); 7.22 (1 H, d, J = 15.5 Hz, = CH); 7.30 (1 H, d, J = 15.5 Hz; = CH); 7.34 (2 H, d, J = 8 Hz); 7.59 (2 H, d, J = 8 Hz).

cis-*3,4,5-Trimethoxy-4′-trifluoromethylstilbene (***7a***)*

Isolated as an oil (48%). NMR: 3.70 (6 H, s, 2 × OMe); 3.90 (3 H, s, OMe); 3.90 (3 H, s, OMe); 6.44 (2 H, s, 2,6-H); 6.63 (1 H, d, J = 13 Hz, = C–H); 6.75 (1 H, d, J = 13 Hz, = C–H); 7.43 (2 H, d, J = 8 Hz); 7.56 (2 H, d, J = 8 Hz). $M^+$, 338 (100%); 323 (M–$CH_3$, 98%).

trans-*3,4,5-Trimethoxy-4′-trifluoromethylstilbene (***7b***)*

Isolated as an oil (43%). NMR: 3.91 (3 H, s, OMe); 3.99 (6 H, s, 2 × OMe); 6.80 (2 H, s, 2,6-H); 7.06 (1 H, d, J = 16.5 Hz, = C–H); 7.17 (1 H, d, J = 16.5 Hz, = C–H); 7.65 (4 H, s, 2′,3′,5′,6′-H). $M^+$, 338 (100%); 323 (M–$CH_3$, 20%).

### *Cell culture*

A P388 mouse leukaemia cell line, together with a multidrug-resistant subline (P388 R8/22) were cultured as described previously (McGown and Fox, 1990). A human ovarian carcinoma cell line (A2780) and its multidrug-resistant subline (A2780/ADR) were grown in RPMI medium supplemented with 10% fetal calf serum, 1% pyruvate and 0.1% insulin. All cell lines were mycoplasma free and >95% viable, as measured by trypan blue exclusion, at the start of experimentation. Cytotoxicity tests were carried out using the

MTT asssay (Edmondson *et al.*, 1988). The $ID_{50}$ concentration was calculated with reference to a standard curve constructed for control cells.

*Cell cycle analysis and determination of mitotic index*

P388 cells were incubated at a concentration corresponding to $10 \times ID_{50}$ of the drug for 24 h before fixation [acetone-ethanol (1:1) as described previously (McGown *et al.*, 1984)]. Mitotic indices were measured by the method of Wolpert-de-Filipes *et al.* (1975) using 5% Giemsa stain. One thousand cells per slide were scored following treatment with the drugs (**1**, 0.02 μM; **3a**, 0.2 μM; **4a**, 0.2 μM; **5a**, 20 μM; **6a**, 241 μM; **7a**, 20 μM; **3b**, 50 μM; **4b**, 20 μM; **5b**, 50 μM; **6b**, 20 μM; **7b**, 50 μM).

*Isolation of tubulin*

The procedure was based on a modification of the method of Miglietta *et al.* (1987). Briefly porcine brain was chilled within minutes of slaughter and homogenised in isolation buffer [0.1 M Mes (2-*N*-morpholinomethansulphonic acid), 1 mM EGTA, 0.5 mM magnesium chloride, pH 6.6] before isolation by two cycles of assembly-disassembly. This was further purified (Hamel and Lin, 1984) by addition of 2 M Mes (pH 6.9) followed by assembly-disassembly, yielding electrophoretically pure tubulin.

*Tubulin assembly-disassembly*

The assembly of microtubules from isolated tubulin was carried out spectrophotometrically at 350 nm and utilised the increase in turbidity which is associated with microtubule formation. Assembly was initiated by temperature increase from 10 to 35°C. The effect of drugs on the increase in light absorption was carried out as described previously (McGown and Fox, 1989). Drugs were dissolved in dimethyl sulphoxide (DMSO) (<4%), which did not affect control assembly.

*Competitive binding assays*

The ability of agents to compete with colchicine for binding to tubulin was examined by a spun column method (Na and Timasheff, 1986). Briefly, tubulin (5 μM) was incubated with test compound and colchicine (10 μM, spiked with [$^3$H]-colchicine, 20 nCi $ml^{-1}$) for 90 min in buffer (0.1 M Mes, 1 mM EGTA, 1 mM EDTA, 1 mM magnesium chloride, pH 6.8). The mixture was loaded on to previously prepared columns of 1 ml of G50 Sephadex (in 40 mM Mes, 40 mM Tris, 1 mM magnesium sulphate, pH 7.5, 11.5 ml $g^{-1}$ Sephadex). These were centrifuged (900 *g*, 2 min) and the eluent analysed by liquid scintillation counting using Ecoscint (National Diagnostics, NJ, USA). When tubulin was not present negligible levels of [$^3$H]colchicine were detected (1 ± 1% of control values) indicating that the free (non-protein bound colchicine) compound is not absorbed by the Sephadex. Therefore, all radioactivity arises from tubulin-bound colchicine. All experiments were performed in triplicate.

*Affinity constant and free energy of binding*

The analyses were carried out based on the method of Prakash and Timasheff (1983) as described previously (McGown and Fox, 1989). The assay utilises the change in protein (tubulin) fluorescence which occurs upon binding of drugs. Analysis of these data yields both the dissociation constant and the free energy of drug-protein binding.

Tubulin (1 μM) was incubated with various drug concentrations (covering drug-protein ratios of between 0.1 and 20 in 0.1 M Mes buffer, pH 6.4) for 90 min at room temperature in the dark. The samples were placed on ice before protein fluorescence was analysed (Shimadzu RF540 fluorimeter $\lambda_{ex} = 275$ nm, $\lambda_{em} = 328$ nm). All experiments were performed in duplicate.

*Immunocytochemistry*

The intracellular distribution of microtubules following treatment with drugs was determined using a modification of the method of Fuller and Brinkley (1975). Studies were carried out on Vero cells because of their large cytoplasms and extensive microtubular networks. Drugs were added (50 μM) and the cells incubated in eight-well chamber slides (NUNC, Naperville, USA) for 40 min at 37°C, followed by fixation in paraformaldehyde (3.5% in PBS) for 10 min. The cells were then permeabilised (50:50 ethanol-acetone, −20°C, 7 min), washed, and the free aldehyde groups reduced by borohydride (5 mg $ml^{-1}$, 3 × 5 min washes). Before analysis by fluorescccence microscopy the slides were then blocked (0.1% bovine serum albumin, 20 mM sodium azide, 40 min, 37°C) and stained in a two-step process with a primary antibody against β-tubulin at 1:20 dilution for 70 min at 37°C followed by visualisation with a fluorescein isothiocyanate (FITC)-conjugated secondary antibody (Boehringer) (50 min, 37°C, 1:20 dilution).

### Results

The stilbenes (**3**-**7**) were prepared in high yield by treating the phosphonium bromide (**2**) with *n*-butyl-lithium in THF followed by the addition of a *p*-substituted benzaldehyde (Scheme). This reaction afforded a closely running mixture of the *cis* and *trans* isomers (**3**-**7**), which were separated by careful chromatography on silica gel. The separated isomers were homogeneous on thin-layer chromatographic plates (silica gel), and proton NMR spectroscopy of each of the isomers indicated their stereochemical and chemical purity. (To avoid photochemical isomerisation the separated isomers were kept in the dark.) Proton NMR spectroscopy was used to establish the stereochemistry of the two separated isomers. In some cases the olefinic protons only provided a singlet when determined in deuterochloroform, but the spectra were resolved using hexadeuteroacetone as solvent. The latter solvent emphasised the coupling of the olefinic protons allowing the stereochemical assignments.

The effects of the *cis*-4′-methyl (**3a**) isomer is shown in Figure 3. From these data a value for the 50% inhibition of tubulin assembly was calculated. These data are summarised in Table I. The maximum concentration used was 50 μM. The most potent agent was combretastatin A-4 (**1**), although the *cis*-4′-methyl and 4′-ethyl derivatives (**3a**, **4a**) also showed inhibition of tubulin assembly. The *trans* isomers (**3b**-**7b**) were all inactive. No *trans* isomer of combretastain A-4 was available to carry similar studies.

The affinity of these analogues for tubulin is shown in Table II. Although combretastatin A-4 (**1**) has the highest affinity for tubulin, all the *cis* analogues (**3a**-**7a**) show strong binding, having dissociation constants in the 0.4-2 μM range. The *trans* isomers, with the exception of the isopropyl

**Figure 3** Effect of the *cis*-methyl analogue (**3a**) on the assembly of isolated tubulin.

**Table I** $IC_{50}$ value for the inhibition of tubulin assembly

| Compound | Cis compound $IC_{50}$ value (μM) | Trans compound $IC_{50}$ value (μM) |
|---|---|---|
| **1** | 2.4 ± 1.4 | >50 |
| **3** | 10.8 ± 2.9 | >50 |
| **4** | 7 ± 3 | >50 |
| **6** | >50 | >50 |
| **7** | >50 | >50 |
| **5** | >50 | >50 |

**Table II** Dissociation constants of the 4′-substituted combretastatins

| Compound | Cis Dissociation constant ($K_d$) μM | Trans Dissociation constant ($K_d$) μM |
|---|---|---|
| **1** | **0.40** ± 0.06 | N/A |
| **3** | 0.75 ± 0.01 | 3.53 ± 0.74 |
| **4** | 1.22 ± 0.08 | 2.76 ± 1.34 |
| **6** | 1.52 ± 0.23 | 83.3 |
| **5** | 0.98 ± 0.16 | 1.03 ± 0.31 |
| **7** | 2.06 ± 0.05 | 2.9 ± 0.4 |

**Table III** Percentage of [$^3$H]colchicine remaining bound to tubulin after drug competition

| Compound | [$^3$H]colchicine (per cent control d.p.m.) drug-protein ratio = 10:1 Cis | Trans |
|---|---|---|
| **1** | **12** ± 1 | N/A |
| **3** | 19.4 ± 2.2 | 77.3 ± 3.6 |
| **4** | 17.5 ± 1.4 | 89.7 ± 5.5 |
| **5** | 55.3 ± 8.5 | 99.7 ± 9.7 |
| **6** | 66.2 ± 8.6 | 94.3 ± 11.6 |
| **7** | 55.1 ± 7.8 | 103.8 ± 10.5 |

**Figure 4** Effect of *cis*- (●) and *trans*-methyl (■) analogues (**3a**,**3b**) on the binding of colchicine to isolated tubulin. ■, Control; ●, 15 μM; ◆, 10 μM; ▲, 5 μM; ×, 2.5 μM.

derivative (**6b**), also showed similar affinities for tubulin. However, the $K_d$ values of the *cis* isomers (**3a**–**7a**) were generally lower than those seen for the *trans* isomers (**3b**–**7b**), particularly in the case of the methyl and ethyl derivatives (**3a**,**4a**), whose $K_d$ values were lower by factors of 8.8 and 6.9 respectively. Little difference is noticeable between the *cis* and *trans* isomers.

The abililty of the *cis*- and *trans*-4′-methyl analogues (**3a**,**3b**) to compete with colchicine for binding to tubulin is shown in Figure 4. Whereas the *cis* isomer (**3a**) can inhibit binding of [$^3$H]colchicine, the *trans* isomer (**3b**) shows much lower activity. A summary of data from all analogues is shown in Table III, and indicates the loss of colchicine binding at a fixed ratio of drug to protein (10:1).

The effects of these analogues on the growth of P388 mouse leukaemia cells and A2780 human ovarian carcinoma cells and their multidrug-resistant sublines (P388R8/22 and A2780/ADR) *in vitro* are shown in Table IV. These data show combretastatin A-4 (**1**) to be the most cytotoxic of the agents tested against both the mouse leukaemia (P388) and human ovarian (A2780) cell lines. This agent is equally potent in the multidrug-resistant cell lines, P388R8/22 and A2780/ADR. The *cis*-methyl and ethyl analogues (**3a**,**4a**) were also highly cytotoxic in all cell lines tested. In general, cytotoxicity decreased with increasing side chain size, with the bulky isopropyl group showing the lowest inhibition of cell growth. The *trans*-isomers (**3b**–**7b**) were much less toxic, and only showed significant growth inhibition at concentrations in the micromolar range.

All *cis* isomers (**3a**–**7a**) tested were found to be capable of causing cell cycle arrest, with cells completing DNA synthesis but being unable to undergo cell division (Figure 5). Evidence of the start of further DNA synthesis, without prior cell division, was observed at later times and was seen as cells with >4*n* DNA content. The *trans* isomers (**3b**–**7b**) were, in general, less effective at inducing a $G_2$/M block, only the trifluoromethyl and ethyl derivatives (**7b**,**4b**) causing $G_2$/M block (Figure 6). The findings of these cell cycle studies are in agreement with the increase in mitotic index seen following treatment with the *cis* isomers (**3a**–**7a**), and also with the *trans*-trifluoromethyl and ethyl isomers (**7b**,**4b**) (Table V).

The intracellular network of microtubules seen in un-

**Table V** Percentage of cells in mitosis following 12 h treatment with drug. Control (untreated) cells showed 2% mitoses

| Compound | Mitoses in P388 cells (%) Cis | Trans |
|---|---|---|
| **1** | 15 | ND |
| **3** | 18 | 1 |
| **4** | 31 | 24 |
| **5** | 13 | 2 |
| **6** | 16 | 9 |
| **7** | 24 | 9 |

**Table IV** Growth inhibition in P388, P388R8/22, A2780 and A2780/ADR cells. Results show mean ± errors of triplicate experiments

| Compound | P388 | P388R8/22 | A2780 | A2780/ADR |
|---|---|---|---|---|
| Cis *isomers* | | | | |
| **1** | 2.6 ± 1.0 | 1.2 ± 0.8 | 0.72 ± 0.23 | 0.84 ± 0.23 |
| **3a** | 9.1 ± 8.1 | 13.4 ± 6.1 | 25.8 ± 9.8 | 23.0 ± 2.0 |
| **4a** | 8.4 ± 4.3 | 11.0 ± 4.0 | 56.5 ± 3.5 | 16.0 ± 3.5 |
| **5a** | ND | ND | 273 ± 78 | 179 ± 21 |
| **6a** | 3800 ± 1600 | 640 ± 120 | 400 ± 120 | 400 ± 30 |
| **7a** | ND | ND | 180 ± 40 | 155 ± 65 |
| Trans *isomers* | | | | |
| **3b** | 31 000 ± 8000 | 17 300 ± 400 | 1800 ± 700 | 9400 ± 150 |
| **4b** | 3800 ± 2900 | 2800 ± 2300 | 4900 ± 900 | 3300 ± 330 |
| **5b** | ND | ND | 2200 ± 1100 | 7000 ± 100 |
| **6b** | >50 000 | >50 000 | >50 000 | >50 000 |
| **7b** | ND | ND | 6600 ± 900 | 11 000 ± 200 |

**Figure 5** The accumulation of cells with ⩾4*n* DNA content following treatment with the *cis* isomers (**3a**–**7a**). ●, Control; ×, **1**; ■, **3a**; ▲, **4a**; ○, **5a**; □, **6a**; △, **7a**.

**Figure 6** The accumulation of cells with ⩾4*n* DNA content following treatment with the *trans* isomers (**3b**–**7b**). ●, DMSO; ■, **3a**; ▲, **4a**; ○, **5a**; □, **6a**; △, **7a**.

treated cells (Figure 7a) is destroyed by incubation with all *cis* isomers (**3a**–**7a**) (Figure 7b–g), resulting in the production of a diffuse staining pattern when compared with the control cells. In contrast, treatment with the *trans* isomers (**3b**–**7b**) does not produce this effect (Figure 7h–l) and the microtubule structures appear similar to the untreated controls (Figure 7a).

### Discussion

The vinca alkaloids are among the most widely used anticancer drugs, showing activity in a number of human malignancies. The mechanism of action of these agents is widely accepted to involve disruption of microtubule-associated processes, particularly the mitotic apparatus. The complexity of these natural or semisynthetic drugs has restricted the development of analogues. The naturally occurring combretastatins are much simpler compounds which are suitable for analogue development via structure-activity studies. We therefore synthesised and tested a number of stilbenes in order to examine the effect of the introduction of an alkyl or fluoroalkyl group in the 4′ position of the stilbene.

These results show that combretastatin A-4 (**1**), the lead compound, binds strongly to tubulin, is a powerful inhibitor of tubulin assembly and is a potent cytotoxic agent *in vitro*. Replacement of the 4′-methoxy group with a short (methyl or ethyl) group together with removal of the 3′-hydroxyl group in the *cis* conformation does not drastically reduce interaction with tubulin. Indeed, although combretastatin A-4 (**1**) was the most potent of the series tested, the *cis*-methyl- and ethyl-stilbenes (**3a**,**4a**) have been shown to interact strongly with tubulin and can inhibit assembly of microtubules, cause disruption of intracellular microtubular structure and inhibit colchicine binding to tubulin, and are cytotoxic to cultured cells. This provides evidence that the 4′-methoxy and 3′-hydroxy groups of combretastatin A-4 (**1**) are not essential for interaction with tubulin.

A summary of these findings is shown in Table VI. Clearly, the stereochemistry of the ethene bridge is very important. The *trans* agents (**3b**–**7b**) do not cause inhibition of tubulin assembly *in vitro*, are much less cytotoxic than the

**Figure 7** Effect of stilbenes (**3**–**7**) on the intracellular organisation of microtubules. (**a**) Control, (**b**) combretastat in A-4 (**1**), (**c**) **3a**, (**d**) **7a**, (**e**) **4a**, (**f**) **5a**, (**g**) **6a**, (**h**) **3b**, (**i**) **7b**, (**j**) **4b**, (**k**) **5b**, (**l**) **6b**.

**Table VI** Comparison of the *cis* and *trans* isomers

| *Property* | Cis | Trans |
|---|---|---|
| Assembly inhibition | **1**>**4a**>**3a**>>**6a**,**5a**,**7a** | All inactive |
| $K_d$ | **1**>**3a**>**5a**>**4a**>**6a**>**7a**<br>Values similar | **5b**>**4b**>**7b**>**3b**>**6b**<br>*Trans*>>*Cis* |
| Colchicine displacement | **1**>**4a**>**3a**>>**7a**,**5a**,**6a**<br>*Cis*>*Trans* | **3b**>**4b**,**6b**,**5b**,**7b** |
| Cytotoxicity (P388) | **1**,**3a**,**4a**>>**6a** | **4b**>**3b**>>**6b** |
| Cytotoxicity (A2780) | **1**>**3a**>**4a**>**7a**>**5a**>**6a**<br>*Cis*>>*Trans* | **3b**,**5b**>**4b**,**7b**>>**6b** |

**Figure 8** Idealised structure of a microtubule-disrupting agent.

corresponding *cis* isomers (**3a**–**7a**) and are not efficient at competing with colchicine for binding to tubulin. Surprisingly, they do bind to tubulin with dissociation constants and free energies of binding which are similar to those of *cis* isomers (**3a**–**7a**). However, their lack of effect on tubulin assembly and colchicine binding is evidence that the *trans* isomers (**3b**–**7b**) bind at sites remote from the colchicine/*cis*-stilbene binding site. Similarly, whereas the *cis* isomers (**3a**–**7a**) were shown to cause disruption to intracellular microtubular networks, the *trans* isomers (**3b**–**7b**) were ineffective (Figure 7).

In general, increasing the length of the substituent chain leads to a reduction in binding to tubulin and cytotoxicity for the more active *cis* isomers (**3a**–**7a**). Interaction with tubulin is tolerant of the replacement of the 4′-methoxy by methyl or ethyl groups, but significant loss of activity is seen with the larger propyl or isopropyl gropus and when the electron-withdrawing trifluoromethyl group is used. It is also interesting to note that, whereas all the *cis* isomers (**3a**–**7a**) caused a $G_2$/M phase block in the cell cycle and an increase in mitotic index, the *trans*-ethyl and -trifluoromethyl isomers (**4b**,**7b**) also showed this effect. This was not reflected in the cytotoxic effects of the *trans* agents (**4b**,**7b**). Why only these two agents (**4b**,**7b**) cause this block is not known, particularly as no disruption of the intracellular microtubular network was observed even at 50 μM. In general, these studies are in agreement with the report by Cushman *et al.* (1992) that the *cis*-methyl and -ethyl derivatives (**3a**,**4a**) inhibit tubulin assembly. This present study further characterises the interaction of agents of this type with tubulin.

The optimal structure for an agent to bind to the colchicine site on tubulin has not yet been fully elucidated. However, this work, in combination with previous studies, can allow us to assign certain structural features to biological activity. The following features have been proposed to be important:

(a) Two (or more) planar rings tilted with respect to each other (McGown and Fox, 1989).
(b) The trimethoxy motif on the A ring is common to a number of agents which are known to interact with tubulin (colchicine, combretastatins, podophyllotoxins, steganacin etc.).
(c) The 2′-hydroxyl group has been implicated in multidrug resistance (MDR) recognition (McGown and Fox, 1989). Omission of this group has been shown to result in molecules which are not involved in the MDR process.
(d) The 3′-hydroxyl group has a relatively small effect on binding to tubulin (Cushman *et al.*, 1992).
(e) The 4′-methoxy group of combretastatin A-4 (**1**) can be replaced with small hydrophobic groups while still retaining significant activity against tubulin.
(f) The ethene bond must be in the *cis* configuration.

These are shown schematically in Figure 8.

In conclusion, we have shown that substitution of small aklyl groups at the 4′ position of combretastatin A-4 (**1**) and loss of the 3′-hydroxyl group does not significantly inhibit the interaction of these agents with tubulin. The results of this study have substantially added to our current knowledge of the structural requirements for tubulin binding.

**Acknowledgements**

This work was supported by the Cancer Research Campaign.

**References**

CUSHMAN M, NAGARATHNAM D, GOPAL D, HE H-M, LIN CM AND HAMEL E. (1992). Synthesis and evaluation of analogues of (Z)-1-(4-methoxyphenyl)-2-(3,4,5-trimethoxyphenyl)ethene as potential cytotoxic and antimitotic agents. *J. Med. Chem.*, **35**, 2293–2306.

EDMONDSON JM, ARMSTRONG LS AND MARTINEZ AO. (1988). A rapid and simple MTT-based assay for determining drug sensitivity in monolayer cultures. *J. Tissue Culture Methods*, **11**, 15–17.

FULLER GM, BRINKLEY BR AND BOUGHTER JM. (1975). Immunofluorescence of mitotic spindles by using monospecific antibodies against bovine brain tubulin. *Science*, **187**, 948–950.

HAMEL E AND LIN CM. (1984). Separation of active tubulin and microtubule associated proteins by ultracentrifugation and isolation of a component causing the formation of microtubule bundles. *Biochemistry*, **23**, 5314–5325.

MCGOWN AT AND FOX BW. (1989). Structural and biochemical comparison of the anti-mitotic agents colchicine, combretastatin A4, and amphethinile. *Anti-Cancer Drug Design*, **3**, 249–254.

MCGOWN AT AND FOX BW. (1990). Differential cytotoxicity of combretastatins A1 and A4 in two daunorubicin-resistant P388 cell lines. *Cancer Chemother. Pharmacol.*, **26**, 79–81.

MCGOWN AT, POPPITT DG, SWINDELL R AND FOX BW. (1984). The effect of vinca alkaloids in enhancing the sensitivity of a methotrexate resistant (L1210/R7/A) line, studied by flow cytometric and chromosome number analysis. *Cancer Chemother. Pharmacol.*, **13**, 47–50.

MIGLIETTA A, GABRIEL L AND GADONI E. (1987). Microtubular protein impairment by pentanal and hexanal. *Cell Biochem. Function*, **5**, 189–192.

NA GC AND TIMASHEFF SN. (1986). Interaction of vinblastine with calf brain tubulin - multiple equilibria. *Biochemistry*, **25**, 6214-6222.

PETTIT GR, SINGH SB, NIVEN ML, HAMEL E AND SCHMIDT JM. (1987). Antineoplastic agents. 124. Isolation, structure, and synthesis of combretastatin A1 and combretastatin B1, potent new inhibitors of microtubule assembly, derived from combretum caffrum. *J. Natl Prod.*, **50**, 119-131.

PETTIT GR, SINGH SB, HAMEL E, LIN CM, ALBERTS DS AND GARIA-KENDALL D. (1989). Isolation and structure of the strong cell growth and tubulin inhibitor combretastatin A4. *Experientia*, **45**, 205-211.

PRAKASH V AND TIMASHEFF SN. (1983). Interaction of vincristine with calf brain tubulin. *J. Biol. Chem.*, **258**, 1689-1696.

WOLPERT DE FILIPES MK, BONO VH, DION RL AND JOHNS DG. (1975). Initial studies on maytansine-induced metaphase arrest in L1210 murine leukemic cells. *Biochem. Pharmacol.*, **24**, 1735-1738.